# On-target and off-target effects of novel orthosteric and allosteric activators of GPR84

**DOI:** 10.1038/s41598-019-38539-1

**Published:** 2019-02-12

**Authors:** Sarah J. Mancini, Zobaer Al Mahmud, Laura Jenkins, Daniele Bolognini, Robert Newman, Matt Barnes, Michelle E. Edye, Stephen B. McMahon, Andrew B. Tobin, Graeme Milligan

**Affiliations:** 1 Centre for Translational Pharmacology, Institute of Molecular, Cell and Systems Biology, University of Glasgow, Glasgow, G12 8QQ United Kingdom; 2 Sosei Heptares, Steinmetz Building, Granta Park, Great Abington, Cambridge, CB21 6DG United Kingdom; 3 Wolfson Centre for Age-Related Diseases, King’s College London, London, SE1 1UL United Kingdom

## Abstract

Many members of the G protein-coupled receptor family, including examples with clear therapeutic potential, remain poorly characterised. This often reflects limited availability of suitable tool ligands with which to interrogate receptor function. In the case of GPR84, currently a target for the treatment of idiopathic pulmonary fibrosis, recent times have seen the description of novel orthosteric and allosteric agonists. Using 2-(hexylthiol)pyrimidine-4,6 diol (2-HTP) and di(5,7-difluoro-1H-indole-3-yl)methane (PSB-16671) as exemplars of each class, in cell lines transfected to express either human or mouse GPR84, both ligands acted as effective on-target activators and with high co-operativity in their interactions. This was also the case in lipopolysaccharide-activated model human and mouse immune cell lines. However in mouse bone-marrow-derived neutrophils, where expression of GPR84 is particularly high, the capacity of PSB-16671 but not of 2-HTP to promote G protein activation was predominantly off-target because it was not blocked by an antagonist of GPR84 and was preserved in neutrophils isolated from GPR84 deficient mice. These results illustrate the challenges of attempting to study and define functions of poorly characterised receptors using ligands that have been developed via medicinal chemistry programmes, but where assessed activity has been limited largely to the initially identified target.

## Introduction

Although a substantial number of G protein-coupled receptors (GPCRs) are the targets of therapeutic medicines and have been extensively studied^1^, this is not the case for many other family members. More than 100 GPCRs remain ‘orphans’ in that the endogenously produced ligands that activate them are either unknown or are not fully accepted^2^ and many more are lacking well-characterised ligands that can be used to help define their function and physiological roles. Despite this, based on links to disease^3^, or phenotypes associated with ‘knock-out’ of the corresponding gene in mouse models, a number of these poorly defined GPCRs are currently considered to offer potential therapeutic opportunities. A case in point is GPR84, where blockade may be effective in idiopathic pulmonary fibrosis^4^ and other fibrotic indications, and where previous studies have assessed whether antagonism of this receptor might be effective in the treatment of ulcerative colitis^5^. Moreover, it has also been suggested that activation of GPR84 may result in effects beneficial for treatment of atherosclerosis^6^.

Shown more than 10 years ago to be activated by medium chain length fatty acids (MCFAs)^7^, this receptor officially remains an orphan^2^. This reflects that the potency/affinity of MCFAs at this receptor is low and that concentrations of circulating MCFAs may not routinely reach levels sufficient to occupy the receptor effectively. In recent times a number of more potent activators of GPR84 have been described. These include 2,5-dihydroxy-3-undecyl-2,5-cyclohexadiene-1,4-dione (embelin)^6^, 6-(octylamino) pyrimidine-2,4(1 H,3 H)-dione (6-*n*-octylaminouracil, 6-OAU)^8–10^ and 2-(hexylthiol)pyrimidine-4,6 diol (2-HTP), which has previously been designated either as ‘compound 1’^11,12^ or ZQ-16^13,14^. Because agonist actions of each of MCFAs, embelin and 2-HTP at human GPR84 are prevented by mutation of arginine172 of the receptor^11^ that acts to co-ordinate the carboxylate of MCFAs, both embelin and 2-HTP are classified as orthosteric agonists, i.e. they bind at the same site as the MCFAs that are notionally the endogenous activators of the receptor. As with many other GPCRs, GPR84 can also be activated by ligands that bind at a distinct site(s). The best studied of these is 3,3′diindoylmethane (DIM)^11,15,16^ which is generated *in vivo* by metabolism of indole-3-carbinol. The effects of DIM are not attentuated by mutation of arginine172^11^ and, therefore, DIM and related molecules are described as allosteric agonists at GPR84. Although not considered as an endogenous agonist, it has been calculated that DIM binds human GPR84 with substantially higher affinity than do MCFAs such as decanoic acid^11^. Progress in generating ligands related to DIM but with higher potency has recently been reported by Pillaiyar *et al*.^16^, with di(5,7-difluoro-1H-indole-3-yl)methane (PSB-16671) the most potent analogue generated^16^. Antagonists of GPR84 are particularly limited, with GLPG1205 and related molecules, including compound 107 (2-([1,4]dioxan-2-ylmethoxy)-9-(3-phenylamino-prop-1-ynyl)-6,7-dihydro-pyrimido[6,1-a]isoquinolin-4-one), being the only reported and studied examples^11,14^. These act as high affinity, but non-competitive, blockers of the actions of both orthosteric and allosteric agonists of GPR84^11^ and GLPG1205 has been used to block effects of GPR84 activation in human phagocytes^14^.

Herein, we set out to establish the detailed pharmacology and usefulness of 2-HTP and PSB-16671 to characterise and define the biology and function of GPR84 in both human and mouse. PSB-16671 produced activation and co-operativity effects that were mediated directly by GPR84 both in cells transfected to express either human or mouse GPR84, and in human and mouse-derived immune cell models. However the ability of PSB-16671 to activate G-proteins in mouse bone marrow-derived neutrophils was instead largely ‘off-target’ and not mediated by GPR84. These conclusions are based on the inability of a GPR84 antagonist that blocked effects of PSB-16671 at mouse GPR84 in both transfected cells and in the macrophage-like RAW264.7 cell line to do so in bone marrow-derived neutrophils, and because the activity of PSB-16671 is preserved in neutrophils from GPR84 knock-out mice. These studies indicate the challenges and dangers of simply transferring outcomes from medicinal chemistry programmes into physiological studies and outcomes for poorly characterised receptors where little is known about the mode of binding of ligands that are described to have potency and affinity at that specific target. Doing so may result in incorrect assignment of functions to such a receptor.

## Results

### Human and mouse GPR84 display similar responsiveness to both orthosteric and allosteric agonists

2-(hexylthiol)pyrimidine-4,6 diol (2-HTP) is a potent agonist of human GPR84^11,13,14^. This ligand has been shown to share an overlapping binding site on human GPR84 with MCFAs such as decanoic acid (C10) that are considered the likely endogenous ligands of the receptor. This is based on effects of both 2-HTP and C10 being eliminated following mutation of arginine172 in the receptor^11^ and because, when added concurrently at submaximal concentrations, these two ligands produce additive, rather than co-operative, responses^11,12^. By contrast, 3,3′diindoylmethane (DIM) and related activators of GPR84 bind to a different site as their function is unaffected by mutation of arginine172^11^ and at sub-maximal concentrations they produce co-operative effects with either MCFAs or 2-HTP^11^. Recently Pillaiyar *et al*.^16^ identified di(5,7-difluoro-1H-indole-3-yl)methane (PSB-16671) as a more potent analogue of DIM at human GPR84.

**Figure 1:**
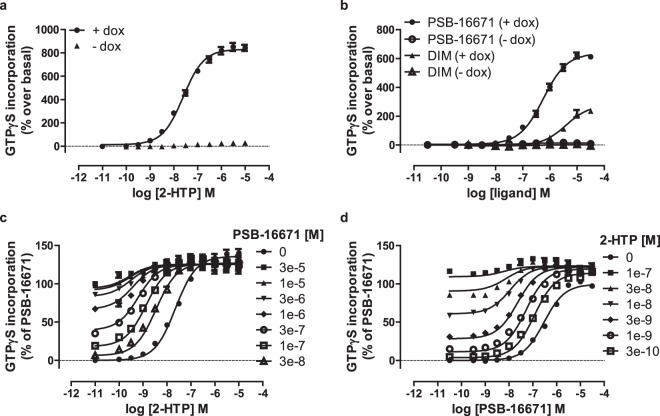
Characterisation of orthosteric and allosteric activators of human GPR84. Flp-In T-REx 293 cells harbouring a human GPR84-Gα_i2_ fusion protein were either untreated (−) or treated with doxycycline (100 ng.ml^−1^, 24 h) (+) to induce expression of the fusion construct. Membranes from these cells were then used to measure binding of [^35^S]GTPγS in response to varying concentrations of 2-HTP (**a**) and either PSB-16671 or DIM (**b**). Equivalent studies measured the effects of various concentrations of PSB-16671 on the potency and efficacy of 2-HTP (**c**) or *vice versa* (**d**). Data represent means ± S.E.M. of combined data from experiments performed on 4 individual membrane preparations. See Table 1 for quantitative analysis.

GPR84 couples selectively to members of the pertussis toxin-sensitive G_i_-G protein family^7,8,11^. Activation of these G proteins is particularly amenable to analysis by measuring regulation of binding of guanine nucleotides and their analogues^17^. Initially, therefore, we examined the ability of 2-HTP to promote the binding of [^35^S]GTPγS in membranes of Flp-In T-REx 293 cells that had been induced to express a human GPR84-Gα_i2_ fusion protein^11^. 2-HTP promoted binding of [^35^S]GTPγS in a concentration-dependent manner with pEC_50_ = 7.54 ± 0.06 (Fig. 1a). Addition of a range of concentrations of PSB-16671 to cell membranes expressing human GPR84-Gα_i2_ showed that this ligand can also activate human GPR84 with, in this case, pEC_50_ = 6.28 ± 0.05 (Fig. 1b). Equivalent studies using DIM confirmed the higher potency of PSB-16671 as originally reported by^16^ (Fig. 1b), but noticeably PSB-16671 also displayed markedly greater efficacy, i.e. greater effect at maximally effective concentrations, than DIM (Fig. 1b). Importantly, no significant stimulation of binding of [^35^S]GTPγS was produced by either 2-HTP or PSB-16671 when these were added to membranes of cells harbouring human GPR84-Gα_i2_ but in which expression of the construct had not been induced (Fig. 1a,b). This demonstrates that both ligands act in an ‘on-target’ manner to directly activate human GPR84. When sub-maximal concentrations of PSB-16671 were co-added with 2-HTP to membranes expressing human GPR84-Gα_i2_ this resulted in enhanced potency of 2-HTP (Fig. 1c), and fitting of such data to an operational model of allosterism^18^ indicated that the extent of co-operativity (α) was greater than 200 fold (log α 2.41 ± 0.17) (Table 1), whilst effects on ligand efficacy were marginal (log β was close to zero) (Table 1). Such data sets also allowed estimation of the binding affinity of 2-HTP at human GPR84 (pKa = 7.0 ± 0.2) and of PSB-16671 (pKa = 6.02 ± 0.13). Reciprocal experiments in which the effects of increasing concentrations of 2-HTP on the potency of PSB-16671 were assessed (Fig. 1d) resulted in similar conclusions and highly similar estimates of the binding affinities of the two ligands (Table 1).

**Table 1:** Binding affinity and co-operativity of ligands as activators of human GPR84.

Agonist^a^	Modulator^b^	logα	logβ	pK_A_^c^	pK_B_^d^
2-HTP	PSB-16671	2.41 ± 0.17	−0.15 ± 0.06	7.0 ± 0.2	6.02 ± 0.13
PSB-16671	2-HTP	2.02 ± 0.11	0.37 ± 0.12	6.1 ± 0.1	7.42 ± 0.07

^a^Agonist is the compound used to generate concentration-response curve.

^b^Modulator is the compound used in defined concentrations.

^c^pK_A_ are values estimated for the agonist.

^d^pK_B_ are values estimated for the modulator.

Data are means ± SEM, n = 4.

Flp-In T-REx 293 cells harbouring a human GPR84-Gα_i2_ fusion protein were treated with doxycycline (100 ng.ml^−1^, 24 h) to induced expression of the fusion protein. [^35^S]GTPγS binding studies were then performed as described in Fig. 1 and binding affinity and cooperativity factors extracted from such data.

**Figure 2:**
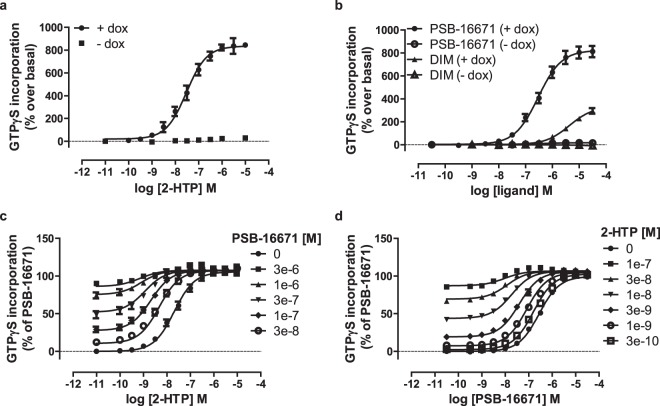
Characterisation of orthosteric and allosteric activators of mouse GPR84. Flp-In T-REx 293 cells harbouring a mouse GPR84-Gα_i2_ fusion protein were either untreated (−) or treated with doxycycline (100 ng.ml^−1^, 24 h) (+) to induce expression of the fusion construct. Membranes from these cells were then used to measure binding of [^35^S]GTPγS in response to varying concentrations of 2-HTP (**a**) and either PSB-16671 or DIM (**b**). Equivalent studies measured the effects of various concentrations of PSB-16671 on the potency and efficacy of 2-HTP (**c**) or *vice versa* (**d**). Data represent means ± S.E.M. of combined data from experiments performed on 4 (**a**) or 5 (**b**) individual membrane preparations. See Table 2 for quantitative outcomes.

As mouse models of GPR84 function and various mouse-derived immune cell lines are central to studies on this receptor we cloned mouse GPR84 from RAW264.7 cells. Following construction into an equivalent Gα_i2_ fusion protein, stable integration into Flp-In T-REx 293 cells, and doxycycline-induced expression of the construct, membrane preparations were again tested for response to, and co-operativity between, 2-HTP and PSB-16671 (Fig. 2). Outcomes were very similar to those observed when using the human orthologue of GPR84, pEC_50_ 2-HTP: 7.52 ± 0.11, PSB-16671: 6.53 ± 0.07, DIM: 5.37 ± 0.08 (Fig. 2, Table 2), and once again DIM acted as a partial agonist when compared to PSB-16671 (Fig. 2).

**Table 2:** Binding affinity and co-operativity of ligands as activators of mouse GPR84.

	Mouse GPR84-Gα_i2_		RAW264.7	
Agonist^a^	2-HTP	PSB-16671	2-HTP	PSB-16671
Modulator^b^	PSB-16671	2-HTP	PSB-16671	2-HTP
logα	2.02 ± 0.11	2.15 ± 0.1	2.4 ± 0.04	1.94 ± 0.03
logβ	0.05 ± 0.09	0.12 ± 0.04	−0.17 ± 0.05	0.33 ± 0.08
pK_A_^c^	6.98 ± 0.17	5.96 ± 0.08	7.0 ± 0.1	6.3 ± 0.15
pK_B_^d^	6.1 ± 0.13	7.2 ± 0.07	6.3 ± 0.1	7.24 ± 0.05

^a^Agonist is the compound used to generate concentration-response curve.

^b^Modulator is the compound used in defined concentrations.

^c^pK_A_ are values estimated for the agonist.

^d^pK_B_ are values estimated for the modulator.

Data are means ± SEM, n = 4.

Either Flp-In T-REx 293 cells harbouring a mouse GPR84-Gα_i2_ fusion protein were treated with doxycycline (100 ng.ml^−1^, 24 h) to induce expression of the fusion protein or membranes were prepared from RAW264.7 cells. [^35^S]GTPγS binding studies were then performed as described in Fig. 2 (mouse GPR84-Gα_i2_ fusion protein) or Fig. 5 (RAW264.7 cells) and estimated binding affinity and cooperativity factors extracted.

### LPS promotes upregulation of the expression and function of GPR84 protein as well as corresponding mRNA

**Figure 3:**
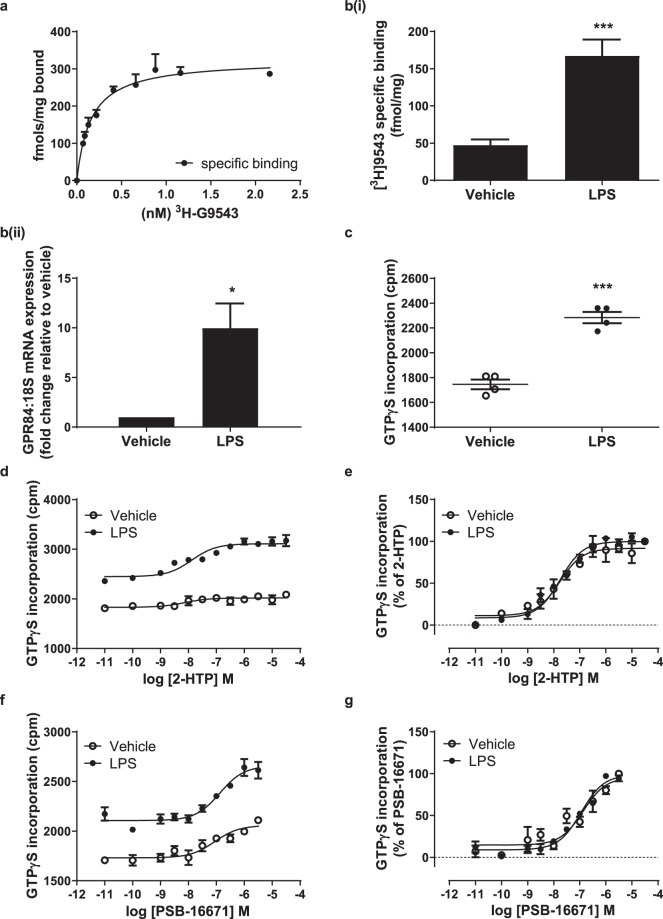
Expression and function of GPR84 in human THP-1 monocytes. THP-1 monocytes were treated with or without LPS (100 ng.ml^−1^) for 24 h. After membrane preparation the specific binding of either varying concentrations of the GPR84 antagonist [^3^H]9543 was measured in membranes from LPS-treated THP-1 cells to allow estimation of both K_d_ and Bmax (**a**) or the specific binding of a single concentration of [^3^H]9543 that was close to 10 times the estimated K_d_ was measured in membranes from both vehicle and LPS- treated cells (**b(i)**). Data in **a** are from a representative experiment of four separate studies in which B_max_ = 293 ± 55 and K_d_ = 0.18 ± 0.02 nM, whilst in **b(i)** they are means ± S.E.M. (n = 7). LPS-treated cells expressed significantly higher amounts of the receptor ***p < 0.001 (two-tail t-test). Equivalent assessments of levels of GPR84 mRNA were conducted (**b(ii)**). Data are means ± S.E.M. (n = 6) *p < 0.05 (two-tail t-test). (**c**) LPS treatment was associated with increased levels of basal binding of [^35^S]GTPγS (n = 4) ***p < 0.001 (two-tail t-test). The ability of varying concentration of 2-HTP (**d**,**e**) and PSB-16671 (**f**,**g**) to promote binding of [^35^S]GTPγS was measured in membranes of both untreated (vehicle) and LPS-treated cells. In (**d**,**f)** data are shown from representative experiments and illustrates the enhanced efficacy of the ligand following LPS treatment, whilst in **e** and **g** data pooled from multiple experiments were normalised and illustrate that the potency of the ligands was not affected by the upregulation of GPR84.

**Figure 4:**
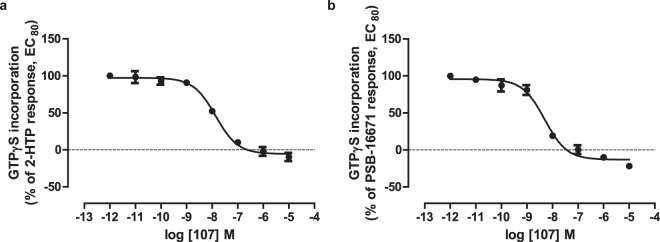
The effects of activators of GPR84 in THP-1 cells are on-target. Membranes were generated from LPS-treated THP-1 cells as in Fig. 3. The ability of varying concentrations of compound 107 to inhibit binding of [^35^S]GTPγS induced by EC_80_ concentrations of either 2-HTP (**a**) and PSB-16671 (**b**) were then measured. Data represent means ± S.E.M. of combined data from experiments performed on 4 individual membrane preparations.

GPR84 is often described as a pro-inflammatory receptor^8^ and is expressed by a range of white cell types and cell lines^7,8,10^. With underpinning pharmacological characterisation in place we turned to explore potential effects of these ligands in human THP-1 monocytes. These cells are known to upregulate expression of mRNA encoding GPR84 in response to activation of toll-like receptor 4 (TLR4) by lipopolysaccharide (LPS)^7,19^. Although this has been suggested to imply a pivotal role of GPR84 in monocyte/macrophage activation and host immune response, nothing is known about how this may reflect or be related to changes in actual expression levels of the receptor. The radiolabelled GPR84 antagonist [^3^H]9543 binds human GPR84 with sub-nM affinity^11^. Saturation [^3^H]9543 binding studies performed on membranes from LPS-treated THP-1 cells confirmed this (K_d_ = 0.18 ± 0.02 nM, (n = 4)) (Fig. 3a). Using a concentration of [^3^H]9543 close to 10 times K_d_ and subsequent correction for predicted receptor occupancy allowed estimation of B_max_ across a range of individual membrane preparations. Membranes prepared from untreated THP-1 cells specifically bound 47 ± 7.5 fmol.mg protein^−1^ [^3^H]9543, whilst after exposure to LPS for 24 hours this increased to 167 ± 22 fmol.mg protein^−1^ (means ± S.E.M., n = 7) (Fig. 3b(i)). This 3.5 fold increase in receptor density compared to a more extensive 10.0 ± 2.5 fold (mean ± S.E.M., n = 6) increase in GPR84 mRNA levels at this time point as assessed using q-RT-PCR (Fig. 3b(ii)). To assess if upregulation of expression of GPR84 in LPS-stimulated THP-1 cells had functional consequences we measured the ability of both 2-HTP and PSB-16671 to promote binding of [^35^S]GTPγS in membranes from these cells. A notable feature in each separate preparation studied was that treatment with LPS resulted in a higher level of basal, ligand-independent binding of [^35^S]GTPγS (Fig. 3c). Moreover, for 2-HTP, the extent of stimulation of binding of [^35^S]GTPγS was markedly higher in membranes derived from LPS-pretreated THP-1 cells than those that were not treated with LPS (Fig. 3d), consistent with the higher measured expression of GPR84 receptor protein. Despite this, no significant increase in potency of 2-HTP was observed (pEC_50_ vehicle = 7.89 ± 0.21, LPS treated = 7.79 ± 0.08) (Fig. 3e), suggesting that even after LPS induced up-regulation no substantial receptor reserve was generated for GPR84. Similar results were observed for PSB-16671 (pEC_50_ vehicle = 6.82 ± 0.12, LPS treated = 6.94 ± 0.07) (Fig. 3f,g). Although these outcomes were consistent with both 2-HTP and PSB-16671 mediating effects via GPR84 in these cells, both ligands are experimental tool compounds with limited broader current characterisation. To further determine if the effects of these ligands in LPS-activated THP-1 cells were actually mediated via GPR84, we assessed if their effects could be blocked by compound 107, which has previously been shown to act as a non-competitive antagonist of human GPR84^11^. For both the activating ligands compound 107 fully blocked their ability to stimulate binding of [^35^S]GTPγS in a concentration-dependent fashion (pIC_50_ versus 2-HTP = 7.86 ± 0.02, versus PSB-16671 = 8.32 ± 0.07) (Fig. 4a,b).

**Figure 5:**
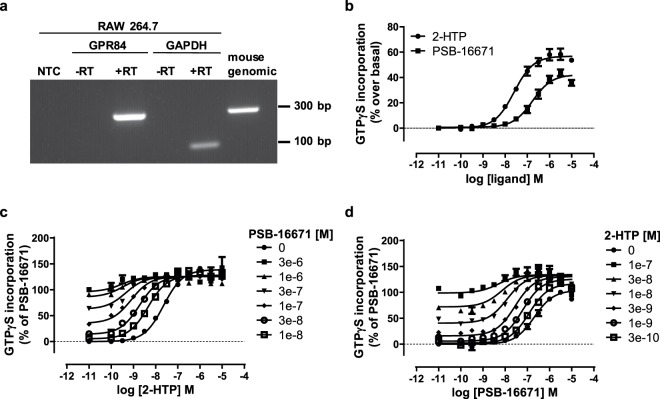
Expression and function of GPR84 in murine RAW264.7 cells. Expression of mRNA encoding GPR84 in RAW 264.7 cells was detected by RT-PCR and compared to amplification of GPR84 from mouse genomic DNA (**a**) (+RT = reverse transcribed, −RT = without reverse transcription, NTC = no template control). Detection of GAPDH was used as a control (predicted amplicon 110 bp). RAW 264.7 cells were treated with LPS (100 ng.ml^−1^, 5 h) and membranes subsequently prepared. The ability of varying concentrations of 2-HTP and PSB-16671 to promote binding of [^35^S]GTPγS were assessed (**b**), as were the effects of various concentrations of PSB-16671 on the potency and efficacy of 2-HTP (**c**) or *vice versa* (**d**). Data represent means ± S.E.M. of combined data from experiments performed on 5 (**b**), 4 (**c**) or 3 (**d**) individual membrane preparations. See Table 2 for quantitative analysis.

The macrophage cell line RAW 264.7 is also a popular model to study immune cell-controlled release of pro-inflammatory mediators. It is, however, a murine-derived cell line, and differences in production of cytokines such as TNFα and IL-1β have been noted compared to human leukocytes^20^. Expression of mRNA encoding mouse GPR84 was clearly detected in these cells using RT-PCR (Fig. 5a) and indeed, as described earlier, we cloned mouse GPR84 from these cells. As expected from the similar calculated binding affinities and G protein activation characteristics of 2-HTP and PSB-16671 at the cloned human and mouse orthologues of GPR84 (Tables 1 and 2) both ligands were able to stimulate binding of [^35^S]GTPγS in membranes of LPS-treated RAW 264.7 cells (pEC_50_ 2-HTP = 7.58 ± 0.08, PSB-16671 = 6.75 ± 0.10) (Fig. 5b) with similar potency as noted for the human GPR84 expressing systems. Moreover, marked co-operative effects were also generated by co-addition of the two ligands (Fig. 5c,d) and estimation of co-operativity and affinity values from these studies were also very similar to those for mouse GPR84 expressed in the Flp-In T-REx 293 cells (Table 2).

### The non-competitive GPR84 antagonist compound 107 has lower affinity at mouse than at human GPR84

**Figure 6:**
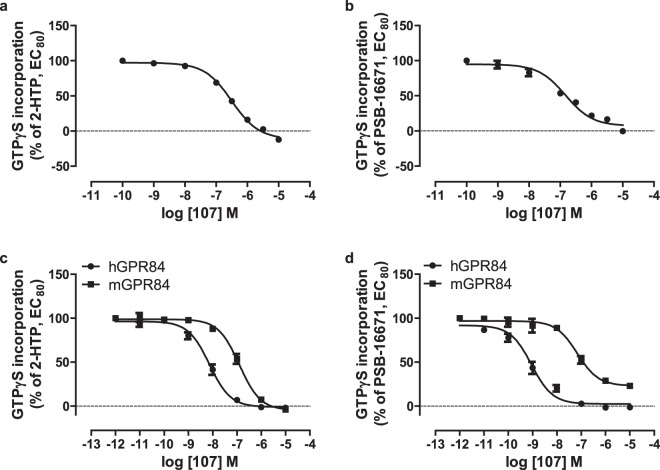
The effects of activators of GPR84 in RAW264.7 cells are on-target. Membranes were generated from LPS-treated RAW264.7 cells (**a**,**b**). These were used in [^35^S]GTPγS binding studies in which the effect of varying concentrations of compound 107 was used to block effects of EC_80_ concentrations of either 2-HTP (**a**) or PSB-16671 (**b**). The ability of compound 107 to block effects of either 2-HTP (**c**) or PSB-16671 (**d**) to promote binding of [^35^S]GTPγS to either human or mouse GPR84-Gα_i2_ fusion proteins expressed in Flp-In T-REx 293 cells is also displayed. Data represent means ± S.E.M. of combined data from experiments performed on 7 (**a**) or 5 (**b**–**d**) individual membrane preparations.

Compound 107 was able to fully block stimulation produced by both 2-HTP and PSB-16671 in membranes from RAW 264.7 cells in a concentration-dependent manner (pIC_50_ versus 2-HTP = 6.50 ± 0.06 and versus PSB-16671 = 6.84 ± 0.09) (Fig. 6a,b). It was also noticeable however that the potency of compound 107 was substantially lower in membranes from RAW 264.7 cells than in equivalent preparations from THP-1 cells. This suggested that compound 107 might have lower affinity at mouse GPR84 compared to the human orthologue. Indeed, when we compared directly the potency of compound 107 to inhibit effects of either 2-HTP or PSB-16671 at cloned human and mouse GPR84 in membranes from Flp-In T-REx 293 cells expressing either human or mouse GPR84-Gα_i2_ fusion proteins compound 107 was between 20 and 75 fold less potent at the mouse orthologue (pIC_50_ human = 8.14 ± 0.06, mouse = 6.90 ± 0.05 versus 2-HTP, and 9.05 ± 0.10 (human) and 7.17 ± 0.10 (mouse) against PSB-16671 respectively) (Fig. 6c,d**)**. As compound 107 is closely related to [^3^H]9543^11^ this lower affinity at mouse GPR84 likely explains why we were unable to obtain direct measures of binding affinity of [^3^H]9543 at mouse GPR84 and, as such, it was not possible, unlike in THP-1 cells, to quantify GPR84 expression levels in RAW 264.7 cells using this radioligand.

### In mouse bone marrow-derived neutrophils G_i_ activation by PSB-16671 is largely independent of GPR84

**Figure 7:**
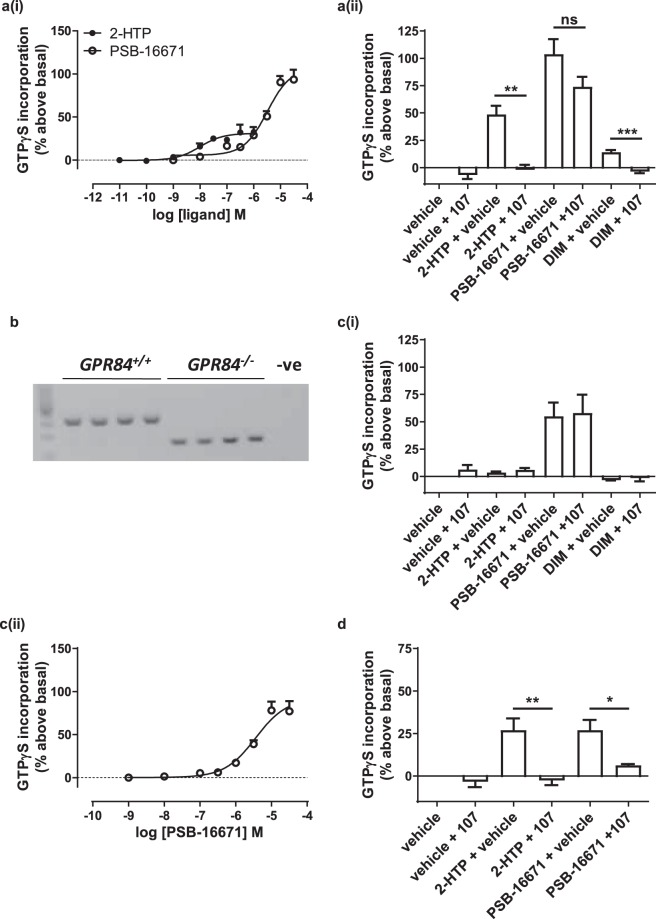
PSB-16671 acts in an off-target, non-GPR84 mediated fashion in mouse bone marrow derived but not human neutrophils. Neutrophils were generated from mouse femur bone marrow taken from wild type (**a**) or GPR84 deficient (**c**) animals. PCR analysis shows genotyping from 4 wild type and 4 GPR84 knock-out mice (**b**). Membranes prepared from these cells were used in [^35^S]GTPγS binding studies to measure effects of varying concentrations of either 2-HTP or PSB-16671 (**a(i)**, **c(ii)**). The ability of compound 107 (10 µM) to block the effects of 2-HTP (1 μM), PSB-16671 (10 μM) or DIM (30 μM) were also assessed (**a(ii)**, **c(i)**). Neutrophil membranes prepared from human whole blood were used in [^35^S]GTPγS binding studies to assess the ability of compound 107 to block the effects of 2-HTP or PSB-16671 (**d**). Data represents means ± S.E.M. of combined data from experiments performed on membrane preparations from 3 individual donors. *p < 0.05, **p < 0.01, ***p < 0.001 relative to agonist only (two-tail t-test).

Despite this limitation we moved forward to assess function of GPR84 in mouse bone marrow-derived neutrophils because the Immunological Genome Project (Immgen) database (http://www.immgen.org/databrowser/index.html) indicates expression of GPR84 mRNA to be highest in these cells. 2-HTP again acted to stimulate binding of [^35^S]GTPγS in membranes isolated from such cells (Fig. 7a). The potency of 2-HTP (pEC_50_ = 7.89 ± 0.12) (Fig. 7a(i)) was similar to those observed in RAW 264.7 cell membrane preparations and this was potentially consistent with its major target in mouse bone marrow-derived neutrophils also being GPR84. However, the potency of PSB-16671 (pEC_50_ = 5.54 ± 0.09) (Fig. 7a(i)) was some 10 fold lower than in RAW 264.7 cell membrane preparations, suggesting that this was not an effect transduced by GPR84. Moreover, although PSB-16671 was not more efficacious than 2-HTP in membranes of either cells transfected to express the mouse GPR84-Gα_i2_ fusion protein (Fig. 2) or in mouse RAW 264.7 cells (Fig. 5), now in membranes from mouse bone marrow-derived neutrophils stimulation of binding of [^35^S]GTPγS by PSB-16671 was much greater than produced by a maximally effective concentration of 2-HTP (Fig. 7a(i)). Although the effect of 2-HTP was completely blocked by compound 107 (Fig. 7a(ii)) the effect of PSB-16671 by contrast was only slightly inhibited by compound 107 (Fig. 7a(ii)). Interestingly, although the parent compound of PSB-16671, DIM, was only able to produce a small fraction of the response generated by PSB-16671, this was instead fully blocked by co-addition of compound 107 (Fig. 7a(ii)).

This combination of observations suggested that a large proportion of the effect of PSB-16671 in mouse bone marrow-derived neutrophils must be an ‘off-target’ effect that is not mediated by GPR84. To test this directly we also isolated bone marrow-derived neutrophils from GPR84 deficient mice (Fig. 7b) and generated membrane preparations. Here, although 2-HTP was unable to stimulate binding of [^35^S]GTPγS (Fig. 7c(i)), PSB-16671 maintained capacity to promote a large increase in [^35^S]GTPγS binding (Fig. 7c(i)) and this was unaffected by the presence of compound 107 (Fig. 7c(i)). In contrast, the effect of DIM observed in wild type bone marrow-derived neutrophils was lacking in those derived from the GPR84 knock-out animals (Fig. 7c(i)), consistent with being mediated by GPR84. Moreover, PSB-16671 stimulated binding of [^35^S]GTPγS in membranes of bone marrow-derived neutrophils of GPR84 deficient mice with potency (pEC_50_ = 5.44 ± 0.14) highly similar to preparations from wild type mice (Fig. 7c(ii)).

### Effects of PSB-16671 in human neutrophils are mediated predominantly by GPR84

**Figure 8:**
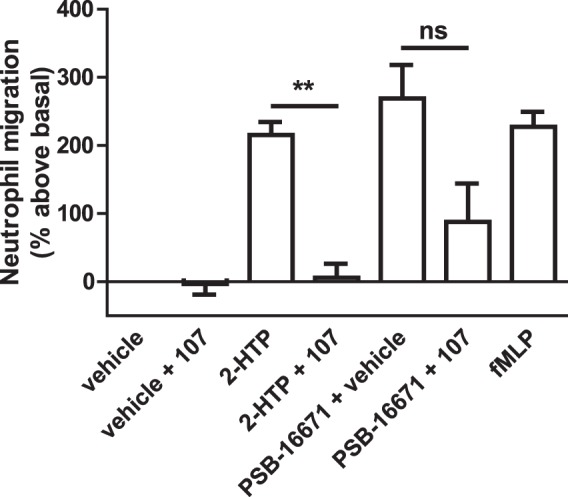
PSB-16671 promotes chemotaxis of mouse bone marrow-derived neutrophils via GPR84. Neutrophils were isolated from wild type mouse femur bone marrow and used in migration studies where the effect of both 2-HTP (100 nM) and PSB-16671 (10 µM), as well as the capacity of pre-treatment with compound 107 (10 µM) to block the effects of these agonists was assessed and compared to the effect of chemotactic agonist fMLP (10 µM). Data represent means ± S.E.M. of combined data from experiments performed on 3 individual membrane preparations. **p < 0.01 relative to agonist only (two-tail t-test).

Based on these observations we also assessed if PSB-16671 might display non-GPR84 mediated effects in human neutrophils. We isolated such cells, generated membrane preparations and again performed a series of [^35^S]GTPγS binding studies. Here both 2-HTP and PSB-16671 were able to promote [^35^S]GTPγS binding, and to a similar extent (Fig. 7d). Moreover, for both the orthosteric and the allosteric agonist their effects were prevented by co-addition of compound 107 (Fig. 7d), consistent with being mediated via GPR84. Finally, although PSB-16671 generated a substantial GPR84-independent increase in binding of [^35^S]GTPγS in mouse bone marrow-derived neutrophils there was potentially a component of this that was sensitive to blockade by the GPR84 antagonist compound 107 (Fig. 7a(ii)). We thus explored if activation of GPR84 promoted migration of these cells. Interestingly, both 2-HTP and PSB-16671 did so (Fig. 8) and to an extent similar to that produced by the chemotactic peptide fMLP (Fig. 8). In this assay compound 107 fully blocked the effect of 2-HTP. By contrast, although compound 107 largely prevented migration in response to PSB-16671 a proportion was not blocked by compound 107 (Fig. 8). This suggests that in mouse bone marrow-derived neutrophils activation of GPR84 does indeed promote cell migration. However, there is also a component of the effect of PSB-16671 on cell migration that is GPR84-independent.

### The effect of PSB-16671 in mouse bone marrow-derived neutrophils is not via GPR35

**Figure 9:**
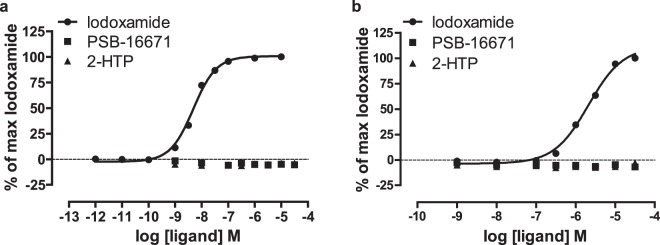
The effect of PSB-16671 in mouse bone marrow-derived neutrophils is not via GPR35. Interactions between eYFP-tagged forms of human GPR35a (**a**) or mouse GPR35 (**b**) and a luciferase-tagged form of β-arrestin 2 was assessed following co-transfection into HEK293 cells and the addition of the indicated concentrations of the GPR35 agonist lodoxamide, 2-HTP or PSB-16671. Results are taken from three independent experiments.

Because GPR84 is phylogenetically related to the receptor GPR35 we assessed whether either PSB-16671 or 2-HTP could also activate mouse GPR35. To do so we used a β-arrestin-2 recruitment assay as this has been the mostly widely used approach to explore the pharmacology of GPR35^21–23^. It has previously been noted that PSB-16671 is unable to activate human GPR35^16^. We confimed this and that 2-HTP was also unable to activate human GPR35, whilst the GPR35 agonist lodoxamide^21^ did so (Fig. 9a), and with high potency (pEC_50_ = 8.33 ± 0.03). Similarly, neither PSB-16671 nor 2-HTP was able to activate mouse GPR35 (Fig. 9b). However, although lodoxamide was able to activate mouse GPR35, it did so with some 500 fold lower potency (pEC_50_ = 5.67 ± 0.01) than at the human orthologue (Fig. 9b).

## Conclusions

Such outcomes illustrate effectively the challenges in efforts to extrapolate the use of ligands that have received limited characterisation during medicinal chemistry structure-activity studies^16^ into broader cell and tissue studies and, potentially, between species orthologues.

## Discussion

Studies on poorly characterised GPCRs, including those activated by fatty acids of varying chain length, are frequently restricted by the limited tool box of pharmacological ligands that are available^24^. This has, for example, greatly hindered efforts to better understand roles of free fatty acid receptor 3^24^. Equally, roles of other GPCRs that have few available pharmcologial tool compounds, such as members of the adhesion class of receptors, are poorly understood despite many being potentially interesting targets in areas such as cancer^25^. Even within the numerically predominant, class A family of receptors that are related to rhodopsin a substantial number remain as ‘orphans’^2^ or have been the subject of a limited number of studies. Whilst such areas of ligand deficit can offer many opportunities for medicinal chemists to produce improved ligands when initial chemical starting points become available, a major challenge is then to understand ligand selectivity and potential ‘off-target’ effects before such compounds can be used with confidence in physiological settings. In recent studies designed to develop analogues of 3-3′-diindoylmethane, Pillaiyar *et al*.^16^, showed that that the higher potency ligand di(5,7-difluoro-1H-indole-3-yl)methane (PSB-16671) was selective for GPR84 in that it did not activate the long chain free fatty acid receptors FFA1 and FFA4 or the phylogenetically related receptor GPR35. Moreover, although DIM is known to have effects at the arylhydrocarbon receptor, PSB-16671 lacked activity at this target^16^.

It is clearly impractical to randomly investigate all possible ‘off-target’ effects of a small molecule ligand, but we were alerted to the possibility that PSB-16671 might produce certain non-GPR84-mediated effects in studies using mouse bone marrow-derived neutrophils. In membranes derived from both Flp-In T-REx 293 cells transfected to express a form of mouse GPR84 and, more importantly, in murine RAW264.7 cells, PSB-16671 and the orthosteric GPR84 agonist 2-HTP appeared to have similar efficacy, because at maximally effective concentrations these two compounds increased levels of [^35^S]GTPγS binding to similar levels. However, in mouse bone marrow-derived neutrophils the stimulation produced by PSB-16671 was markedly higher than produced by 2-HTP. To examine this more directly we assessed whether effects of both 2-HTP and PSB-16671 would be blocked by the non-competitive GPR84 antagonist compound 107, which has previously been shown to antagonise effects of both the MCFA decanoic acid and of DIM at human GPR84^11^. However, although compound 107 did block the effect of 2-HTP almost completely at 10 μM it only marginally inhibited the effect of PSB-16671 in membranes of mouse bone marrow-derived neutrophils. In previous studies we have shown that compound 107 has high affinity at human GPR84 but this has not previously been assessed at the mouse orthologue. When we compared directly the potency of compound 107 to antagonise effects of either 2-HTP or PSB-16671 at human versus mouse GPR84 in cells in which the receptor was expressed as the corresponding Gα_i2_ fusion protein, it was clear that although compound 107 is, indeed, an effective antagonist of the mouse receptor, it is substantially less potent than at the human orthologue. Moreover, although some 20 fold less potent in blocking effects mediated by the orthosteric agonist 2-HTP at the mouse receptor this was even more extensive when considering PSB-16671 which, as an allosteric agonist at both human and mouse GPR84, binds at a different site than 2-HTP. These were important observations and require consideration because antagonists at certain poorly characterised receptors can display very marked selectivity between species orthologues. For example, CID-2745687 (methyl-5-[(tert butylcarbamothioylhydrazinylidene)methyl]-1-(2,4-difluorophenyl)pyrazole-4-carboxylate) is described in both academic publications and supplier catalogues as an ‘antagonist of GPR35′. However, although it is indeed a high affinity antagonist of human GPR35^24^ it has been shown to lack activity at both the rat and mouse orthologues^23^. Similarly, although the clinically trialed FFA2 receptor antagonist GLPG0974 is a high affinity antagonist of the human receptor, this compound also lacks significant activity at rodent orthologues^26^.

To further validate the non-GPR84 mediated effect of PSB-16671 in mouse bone marrow- derived neutrophils we isolated such cells and generated membranes from a GPR84 knock-out mouse line^27^. Here, although 2-HTP lacked activity, PSB-16671 produced stimulation of [^35^S]GTPγS binding almost as effectively as in samples from wild type mice and, once more, this effect was not blocked by compound 107. It was also notable that the potency of PSB-16671 in membranes of bone marrow-derived neutrophils from both wild type and GPR84 deficient mice was almost 10 fold lower than in membranes from RAW264.7 cells, suggesting that it has a further molecular target at which it displays somewhat lower affinity than at mouse GPR84. This illustrates the importance of performing careful pharmacological studies in which the effects of poorly characterised ligands are measured across a range of concentrations, and not simply at a single high concentration. The non-GPR84 molecular target of PSB-16671 in mouse bone marrow-derived neutrophils remains unclear. As the [^35^S]GTPγS binding assay reports on activation of heterotrimeric G proteins, and predominantly on members of the G_i_-subfamily^17^, the site is likely to be a highly expressed G_i_-coupled GPCR. Database information, e.g. the Immgen database (http://www.immgen.org/databrowser/index.html) indicates that such cells express a wide range of GPCRs, with various chemokine receptors, e.g. CXCR4, CXCR2 and CCR1, as well as other receptors associated with chemotaxis e.g. formyl peptide receptor 1 (FPR1) and the complement component 5a receptor 1 (C5a_1_), being particularly highly expressed. However, a number of orphan GPCRs including GPR27, GPR64, GPR107 and GPR141 are also highly expressed at the mRNA level. In time it will be interesting to explore whether PSB-16671 is able to activate one of more of these receptors, either as an orthosteric or an allosteric agonist.

Similarly, efforts have been made to catalogue GPCR expression in RAW264.7 cells^28^ and phorbol ester-stimulated THP-1 cells^29^ and assessment of such profiles may help define the range of potential targets for activation by PSB-16671 that should be prioritised.

A further key feature of these studies was the ability to measure directly levels of expression of GPR84 in human tissues and human-derived cells. Although it is well appreciated that treatment of cell lines such as THP-1 monocytes with TLR4 receptor activators such as LPS results in a time-dependent upregulation of mRNA encoding GPR84^7,10^ it has not previously been possible to assess how this might relate to altered levels of expression of the receptor itself. Herein, by using the specific binding of [^3^H]9543 we were able to do so. Interestingly, although levels of GPR84 receptor protein were upregulated some 3.5 fold and this resulted in higher G protein activation in response to maximally effective concentrations of either 2-HTP or PSB-16671, such treatment did not enhance the potency of either of these ligands. One potential means to improve biological function of an agonist ligand is to increase levels of its receptor in response to a challenge or stress such that a maintained concentration of the endogenous agonist now produces greater effect. However, these results indicate that the LPS-induced upregulation did not generate a marked GPR84 receptor reserve in THP-1 cells. As such, whilst the effect of LPS is likely to allow greater levels of maximal signal from endogenous activators of the receptor, it will not result in improved sensitivity of the cell to such activators.

Recently, clinical trials with ligands that have affinity at GPR84 have explored the potential of blocking this receptor for the treatment of both ulcerative colitis^5^ and idiopathic pulmonary fibrosis^4^,^30^. Furthermore, suggestions that activation of GPR84 might have beneficial effects in atherosclerotic conditions^6^ have begun to provide greater focus on this receptor. In recent times a number of distinct compounds with agonist^6,12,13,31^ or positive allosteric modulator (PAM)-agonist^16^ activation of GPR84 have been decribed and substantial structure-activity relationships of some of these compounds have been illustrated^6,12,13,16,31^. However, before using compounds from such series to claim direct evidence on further roles for GPR84 and its potential therapeutic indications, a better understanding of potential off- target effects are clearly required.

## Methods

### Materials

PSB-16671^16^, compound 104 (9-(5-cyclopropyl-[1,2,4]oxadiazol-3-ylmethoxy)-2-((R)-1-[1,4]dioxan-2-ylmethoxy)-6,7-dihydro-pyrimido[6,1-a]isoquinolin-4-one), compound 107 (2-([1,4]dioxan-2-ylmethoxy)-9-(3-phenylamino-prop-1-ynyl)-6,7-dihydro-pyrimido[6,1-a]isoquinolin-4-one)^11^ and [^3^H]9543^11^ were provided by Laurent Saniere (Galapagos NV). 2-(hexylthiol)pyrimidine-4,6 diol (2-HTP) was provided by Trond Ulven, University of Copenhagen.

### GPR84 knock-out mice

GPR84 (GenBank accession number NM_030720, Ensembl identification number ENSMUSG00000063234) KO mice^27^ were provided by Deltagen under a GlaxoSmithKline license agreement. Targeting replaced 257 bp of coding sequence with an IRES?LacZ?poly(A) expression cassette and a positive selection cassette that contains the neomycin phosphotransferase gene driven by the PGK promoter (Neo). The insertion of the LacZ IRESLacZ introduces a premature translational stop signal that deletes the first three predicted transmembrane domain sequences. Disruption of the GPR84 gene was confirmed using PCR and standard agarose gel electrophoresis using the following primers: gene specific (endogenous), 5′-CAGATGCCAACTTCTCCTGCTA-3′; gene specific (targeted, endogenous), 5′-GAGGTAGCGTCCTAGAGCAAT-3′; and Neo (targeted), 5′-GCCTCTGTTCCACATACA-3′.

Mice forming the initial breeding pairs were supplied by GlaxoSmithKline, which consisted of heterozygous (HET) F1 offspring from WT and KO breeding. HET pairs were bred in-house from 8 weeks old to produce litters of mixed genotypes according to Mendelian genetics.

### Animal maintenance

Both wild type C57BL/6 (Glasgow) and GPR84 knock out (London) mice were fed *ad libitum* with a standard mouse chow diet. Male and female animals at 8–15 weeks old were used. Animals were cared for in accordance with national guidelines on animal experimentation. All animal experiments were conducted under appropriate home office licences. Experiments were conducted under Establishment licence number (PEL) 70/2901 (Kings College) and 70/8473 (Glasgow).

### Cloning of mouse GPR84

Mouse GPR84 was cloned from the mouse leukaemic macrophage cell line RAW 264.7. RNA was extracted from RAW 264.7 cells using RNeasy mini Kit (Qiagen) followed by cDNA synthesis using QuantiTect Reverse Transcription Kit (Qiagen) according to the manufacturer’s instructions. Using this cDNA as template, the FLAG epitope tag (amino acid sequence DYKDDDDK) was incorporated at the N-terminus of mouse GPR84 by PCR reaction using the following primers:

Mouse GPR84 sense: 5′ CAT GTT GGA TCC GCC ACC ATG GAC TAC AAG GAC GAC GAT GAT AAG TGG AAC AGC TCA GAT GCC AAC 3′.

Mouse GPR84 antisense: 5′ CAT GTT GCG GCC GC G ATG GAA CCG GCG GAA ACT CTG 3′.

The sequences corresponding to BamHI and NotI sites required for cloning are underlined.

The resulting PCR amplified product, FLAG-mouse (m)GPR84 was then subcloned in-frame between the BamHI and NotI sites of an eYFP-pcDNA5/FRT/TO plasmid ultimately generating FLAG-mGPR84-eYFP-pcDNA5/FRT/TO construct. The identity of the construct was then confirmed by DNA sequencing. The FLAG-human (h)GPR84-Gα_i2_ fusion protein construct was cloned in pcDNA5/FRT/TO expression vector as described previously^11^. For generation of the FLAG-mGPR84-Gα_i2_ fusion construct, at first the internal BamHI site of Gα_i2_ of FLAG-hGPR84-Gα_i2_ was silently mutated by site directed mutagenesis using the QuikChange method (Stratagene, Cheshire, UK) using the following primers:

C636T Gα_i2_ Forward: 5′ GAGCGGAAGAAGTGGATTCACTGCTTTGAGGGTG 3′.

C636T Gα_i2_ Reverse: 5′ CACCCTCAAAGCAGTGAATCCACTTCTTCCGCTC 3′.

The FLAG-mGPR84-Gα_i2_ fusion protein construct was then generated by replacing human GPR84 of FLAG-hGPR84-C636TGα_i2_ pcDNA5/FRT/TO plasmid with the sequence corresponding to mouse GPR84 using BamHI and Not1 restriction enzymes.

### RNA extraction and gene expression analysis

RNA was extracted from THP-1 cells using an RNeasy kit (Qiagen). Between 1000–2000 ng of RNA was reverse-transcribed using the High Capacity cDNA Reverse Transcription kit (Applied Biosystems). qPCR was performed with an Applied Biosystems ABI-PRISM 7900HT Sequence Detection System. Gene expression was normalised to eukaryotic 18 S ribosomal RNA using qPCR master mix (Applied Biosystems) and the following TaqMan® Gene Expression Assays (Applied Biosystems): GPR84 (Hs00220561_m1) and eukaryotic 18 S rRNA (Hs99999901_s1). GPR84 expression in RAW 264.7 cells was confirmed by RT-PCR using the primers F-5′-AGGTGACCCGTATGTGCTTC-3′ and R-5′-ACTCTGGTTCCGGATGTTTG-3′. For GAPDH the primers used for RT PCR were:

F-5′-TTGATGGCAACAATCTCCAC-3′ and R-5′-CGTCCCGTAGACAAAATGGT-3′.

Anticipated cDNA amplicon size:110 bp. Images shown in Fig. 7 were acquired using Gene Genius Bio Imaging System from Syngene and GeneSnap v7.12.

### Expression of human and mouse GPR84-Gα_i2_ fusion proteins

Doxycycline inducible Flp-In T-REx-293 stable cell lines expressing FLAG-hGPR84-Gα_i2_ and FLAG-mGPR84-Gα_i2_ constructs were generated by co-transfection of the receptor construct of interest and integration plasmid pOG44(1:9) into Flp-In T-REx-293 cells using 1 mg/ml of cationic DNA complexing agent polyethyleneimine (PEI, MW-25000). Flp-In T-REx-293 cells were maintained in Dulbecco’s modified Eagle’s Medium (DMEM) without sodium pyruvate (Invitrogen) supplemented with 10% fetal bovine serum (FBS), 1% penicillin/streptomycin and 10 µg/ml blasticidin at 37 °C in 5% CO_2_ humidified atmosphere. The cells were transfected at 60 to 80% confluency with a total of 8 µg DNA (0.8 µg receptor of interest in pcDNA5/FRT/TO vector and 7.2 µg of pOG44 plasmid vector) and PEI (ratio 1:6 DNA/PEI), each diluted in 150 mM sterile NaCl, pH 7.4. The mixture of DNA complexes and PEI were incubated at room temperature for 10 minutes and then added to the cells. Stably transfected cells were selected by changing medium to medium containing 200 µg/ml hygromycin B 48 hours after transfection. After isolation of stable transfectants, expression of the receptor construct was induced on demand by treating the cells with 100 ng/ml of doxycycline for 24 hours.

### Cell culture

THP-1 monocytes were maintained at a density of between 1 × 10^5^– 8 × 10^5^ cells/ml in RPMI-1640 (Invitrogen) supplemented with 10% (v/v) heat inactivated FBS, 1% penicillin/streptomycin mixture and 2 mM L-glutamine at 37 °C in a 5% CO_2_ humidified atmosphere. Cells seeded at a density of 4 × 10^5^ cells/ml were exposed to lipopolysaccharide (LPS) (100 ng/ml) (Sigma, Dorset, UK) for 24 h prior to RNA extraction or membrane preparation. RAW 264.7 mouse macrophages were maintained in DMEM (with 4.5 g/L D-glucose, 0.11 g/L sodium pyruvate) supplemented with 10% heat inactivated FBS, 1% penicillin/streptomycin and 2 mM L-glutamine at 37 °C and 5% CO_2_ humidified atmosphere. To upregulate GPR84 expression, RAW 264.7 cells were treated with 100 ng/ml of LPS for 5 h prior to membrane preparation. Flp-In™ T-REx™-293 cells (Invitrogen) were maintained in Dulbecco’s modified Eagle’s medium without sodium pyruvate (Invitrogen), supplemented with 10% (v/v) heat inactivated FBS, 1% penicillin/streptomycin mixture, and 10 µg/ml blasticidin at 37 °C in a 5% CO_2_ humidified atmosphere. Expression of the appropriate construct from the Flp-In™ T-REx™ locus was induced by treatment with 100 ng/ml doxycycline for 24 h.

### Membrane preparation

Membranes were generated from THP-1 or RAW 264.7 cells in the presence or absence of LPS stimulation or Flp-In™ T-REx™-293 cells following doxycycline treatment to induce receptor expression. Cells were centrifuged at 3000 rpm for 5 min at 4 °C. Pellets were resuspended in TE buffer (10 mM Tris-HCl, 0.1 mM EDTA; pH 7.5) containing a protease inhibitor mixture (Roche, Applied Science, West Sussex, UK) and homogenised with a 5 ml hand-held homogeniser. This material was centrifuged at 1500 rpm for 5 min at 4 °C and the supernatant was further centrifuged at 50000 rpm for 45 min at 4 °C. The resulting pellet was resuspended in TE buffer and protein content assessed using a BCA protein assay kit (Pierce, Fisher Scientific, Loughborough, UK).

### Isolation of neutrophils from mouse bone marrow

Tibiae and femurs of GPR84 knock-out and wild type mice were removed and freed of soft tissue. Both epiphyses were removed using sterile scissors and bones were flushed with a syringe filled with ice-cold RPMI 1640. Bone marrow suspension was passed through a 100 µm cell strainer and collected in a sterile 15 mL polypropylene tube for centrifugation at 300 *g* for 5 min at 4 °C. The resulting pellet was resuspended in PBS containing 1% FBS and 2 mM EDTA. Neutrophils were isolated using the EasySep™ Mouse Neutrophil Enrichment Kit according to manufacturer’s instructions (STEMCELL Technologies UK Ltd, Cambridge, UK).

### Isolation of neutrophils from human whole blood

Peripheral blood samples were obtained from normal healthy donors following protocols approved by the University of Glasgow, College of Medical, Veterinary and Life Sciences ethics committee. Donors provided written informed consent. Neutrophils were then isolated from human whole blood using the MACSxpress® Neutrophil Isolation Kit according to the manufacturer’s instructions (Miltenyi Biotec, Bergisch Gladbach, Germany).

### Neutrophil migration assay

After isolation, neutrophils were immediately resuspended in RPMI 1640 containing 0.5% fatty acid-free bovine serum albumin. Test compounds were prepared at the indicated concentrations in the same buffer and added at the bottom of a 96-well plate (Sigma-Aldrich). Inserts were then mounted to the plate and neutrophils were added (3000 cells/μL). Cells were incubated at 37 °C for 1.5 h and migrated cells were then collected and ATP content was assessed using ATPlite Luminescence Assay System® according to manufacturer instructions (Perkin Elmer, Buckinghamshire, UK).

### Preparation of neutrophil membranes

Neutrophils were resuspended in 50 mM Tris HCL, 10 mM MgCl_2_ pH 7.4, and centrifuged at 11,000 *g* for 15 min at 4 °C. The supernatant was discarded and pellet was resuspended in 50 mM Tris HCl, pH 7.4 and centrifuged at 11,000* g* for 15 min at 4 °C. The resulting pellet was resuspended in 50 mM Tris-HCl and the protein concentration was determined using a BCA protein assay kit (Expedeon, Cambridge, UK).

### Radioligand binding assay

[^3^H]G9543 is an analogue of compounds 104 and 107 as previously described^11^. Because [^3^H]G9543 binds to human GPR84 with sub nM affinity^11^ most assays were carried out using a close to saturating concentration (2 nM) of [^3^H]G9543, binding buffer (PBS with 0.5% fatty-acid free bovine serum albumin; pH 7.4) in a total assay volume of 500 µl in glass tubes. Binding was initiated by the addition of THP-1 membranes (5 µg of protein per tube). Assays were performed at 25 °C for 1 h before termination by the addition of ice-cold PBS and vacuum filtration through GF/C glass filters using a 24-well Brandel cell harvester (Alpha Biotech, Glasgow, UK). Each reaction tube was washed 3 times with ice-cold PBS. The filters were allowed to dry and then placed in 3 ml of Ultima Gold™ XR (PerkinElmer Life Sciences, Beaconsfield, UK). Radioactivity was quantified by liquid scintillation spectroscopy. Specific binding was defined as the difference between binding detected in the presence and absence of 1 µM compound 104^11^. Correction for occupancy allowed calculation of B_max_. In some studies a range of concentrations of [^3^H]G9543 was used to allow estimation of K_d_ of the ligand and direct measurement of B_max_.

### [^35^S]GTPγS incorporation assay

Prepared membrane protein (5 µg THP-1, RAW-264.7, 3 µg Flp-In T-REx 293 cells) was incubated in assay buffer (20 mM HEPES, 5 mM MgCl_2_, 160 mM NaCl, 0.05% fatty-acid-free bovine serum albumin; pH 7.5) containing the indicated ligand concentrations. In experiments designed to assess inhibition of agonist stimulation, membrane preparations were pre-incubated with antagonist compound 107 for 15 min at room temperature prior to addition of 2-HTP or PSB-16671. In experiments designed to assess potential allosteric interactions between PSB-16671 and 2-HTP, both ligands were added at the same time to the membrane preparation. The reaction was initiated by addition of [^35^S]GTPγS (50 nCi per reaction) with 1 µM GDP, and incubated at 30 °C for 60 min. The reaction was terminated by rapid vacuum filtration through GF/C glassfibre filter-bottom 96-well microplates (PerkinElmer Life Sciences, Beaconsfield, UK) using a UniFilter FilterMate Harvester (PerkinElmer Life Sciences, Beaconsfield, UK). Unbound radioligand was removed from filters by three washes with ice-cold PBS. MicroScint-20 (PerkinElmer Life Sciences, Beaconsfield, UK) was added to dried filters, and [^35^S]GTPγS binding was quantified by liquid scintillation spectroscopy.

### β-arrestin-2 interaction assays

To assess potential agonist effects of GPR84 ligands at the phylogenetically related receptor GPR35, C-terminally enhanced yellow fluorescent protein (eYFP)-tagged forms of either human GPR35a or mouse GPR35 were co-transfected with a luciferase tagged form of β-arrestin-2 into HEK293 cells as described previously^21^. 24 hours later cells were treated with varying concentrations of the GPR35 agonist lodoxamine^21^ or with 2-HTP or PSB-16671. Bioluminescence resonance energy transfer was then measured 5 minutes after addition of the luciferase substrate coelenterazine-h as in^21^.

### Analysis of ligand affinity/co-operativity

To calculate the allosteric parameters data obtained from allosterism experiments were analysed using an operational model of allosteric modulation described previously^18,32^. The general version of this model is denoted by the following equation:

$${\rm{E}}=\frac{{E}_{m}({\tau }_{A}[A]{({K}_{B}+\alpha \beta [B]+{\tau }_{B}[B]{K}_{A})}^{n}}{([A]{K}_{B}+{K}_{A}{K}_{B}+[B]{K}_{A}+\alpha [A]{[B]}^{n}+({\tau }_{A}{[A({K}_{B}+\alpha \beta [B])+{\tau }_{B}[B]{K}_{A})}^{n}}$$

where, E is the measured response, [A] and [B] are the orthosteric and allosteric ligand concentration at equilibrium, respectively; K_A_ and K_B_ represent the binding affinities of the two ligands to the receptor; α is the binding cooperativity factor reflecting the allosteric effect on binding affinity of the orthosteric agonist to the receptor, β is the activation cooperativity factor denoting the empirical measure of the allosteric effect on orthosteric efficacy. The values of τ_A_ and τ_B_ measure the ability of the orthosteric and allosteric ligand, respectively to directly activate the receptor. E_m_ is the maximum system response and n denotes the slope factor of the transducer function. To fit the allosterism data globally through this equation, E_m_ and n values were always constrained and other parameters (K_A_, K_B_, α, β, τ_A_ and τ_B_) were estimated.

## Supplementary information

Supplementary Figure 5 full length gel

## Data Availability

The datasets generated and analysed during the current study are available from the corresponding author on reasonable request.
