# Supplementary material for: On-target and off-target effects of novel orthosteric and allosteric activators of GPR84
Source: Sci Rep. 2019 Feb 12;9:1861. doi: 10.1038/s41598-019-38539-1 (PMC6372602; doi:10.1038/s41598-019-38539-1)

On-target and off-target effects of novel orthosteric and allosteric activators of GPR84

Sarah Mancini, Zobaer Al Mahmud, Laura Jenkins, Daniele Bolognini, Robert Newman, Matt Barnes, Michelle E. Edye, Stephen B. McMahon, Andrew B. Tobin and Graeme Milligan

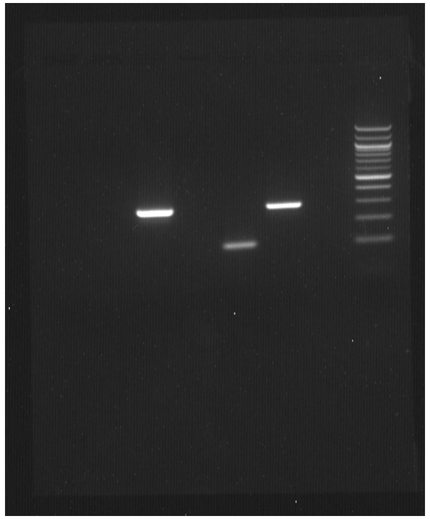

Supplement: Supplementary file 1 — Supplementary Figure 5 full length gel [file 41598_2019_38539_MOESM1_ESM.pdf]
